# Supplementary material for: A Multiclass Mechanistic Model of Vaccine-Induced Humoral Responses
Source: bioRxiv. 2026 Oct 20:2026.10.14.759230. Preprint. [Version 1] doi: 10.64898/2026.10.14.759230 (PMC13738377; doi:10.64898/2026.10.14.759230)
Supplement: Supplementary file 1 [file NIHPP2026.10.14.759230v1-supplement-1.pdf]

bioRxiv preprint doi: https://doi.org/10.64898/2026.10.14.759230; this version posted October 20, 2026. The copyright holder for this preprint (which was not certified by peer review) is the author/funder, who has granted bioRxiv a license to display the preprint in perpetuity. It is made available under a CC-BY 4.0 International license.

Supplementary Materials for

A Multiclass Mechanistic Model of Vaccine-Induced Humoral Response

Sarafa A. Iyaniwura[1*], Bryan T. Mayer[1], William O. Hahn[1,2], Soo Young Kim[1], Yunda Huang[1,3,4], and Ollivier Hyrien[1,4,5†]

[1]Vaccine and Infectious Disease Division, Fred Hutchinson Cancer Center, Seattle, WA, USA.
[2]Department of Medicine, Division of Allergy & Infectious Diseases, University of Washington, Seattle, WA, USA.
[3]Department of Global Health, University of Washington, Seattle, WA, USA.
[4]Public Health Sciences Division, Fred Hutchinson Cancer Center, Seattle, WA, USA.
[5]Department of Biostatistics, School of Public Health, University of Washington, Seattle, WA, USA.

Corresponding authors: *iyaniwura@fredhutch.org & †ohyrien@fredhutch.org

Supplementary Materials

SM1. The G001 model

Immunogen dynamics in the injection site:

$$I_S(t) = \sum_{j=1}^{2} I_{Sj}\, g_j(t)\,, \tag{S1}$$

where $g_j(t) = 0, t < t_j$ and $g_j(t) = e^{-\rho_s(t-t_j)}, t \geq t_j$, with $\rho_s > 0, j = 1, 2$.

Immunogen dynamics in the lymphatic compartment:

$$\begin{aligned}\frac{dI_L}{dt} = \sum_{j=1}^{2} \rho_{sj}\, I_{Sj}\, g_j - \left(c_l + \rho_l + \sum_{i=1}^{3} \frac{\pi_i\, A_i^{\alpha_a}}{A_i^{\alpha_a} + \kappa_a^{\alpha_a}}\right) I_L - \frac{\tau\, \gamma_n\, I_L^{\alpha_n}}{I_L^{\alpha_n} + \kappa_n^{\alpha_n}} N \\ - \frac{\tau\, I_L^{\alpha_m}}{I_L^{\alpha_m} + \kappa_m^{\alpha_m}} \sum_{i=1}^{3} \gamma_{mi}\, M_i - \frac{\tau\, \zeta_a\, I_L^{\alpha_{na}}}{I_L^{\alpha_{na}} + \kappa_{na}^{\alpha_{na}}} N_a\,,\end{aligned} \tag{S2}$$

Immunogen dynamics in the germinal center:

$$\frac{dI_G}{dt} = \rho_l I_L - \vartheta \sum_{i=1}^{3} \lambda_i\, v_i G_i - c_G I_G\,, \tag{S3}$$

Vaccine-specific naïve B-cell dynamics:

$$\frac{dN}{dt} = \Lambda_n - \left(\frac{\gamma_n\, I_L^{\alpha_n}}{I_L^{\alpha_n} + \kappa_n^{\alpha_n}} + \delta_n\right) N\,, \tag{S4}$$

bioRxiv preprint doi: https://doi.org/10.64898/2026.10.14.759230; this version posted October 20, 2026. The copyright holder for this preprint (which was not certified by peer review) is the author/funder, who has granted bioRxiv a license to display the preprint in perpetuity. It is made available under a CC-BY 4.0 International license.

Activated-naïve B-cell dynamics:

$$\frac{dN_a}{dt} = \frac{\gamma_n I_L^{\alpha n}}{I_L^{\alpha n} + \kappa_n^{\alpha n}} N + \left(\frac{\zeta_a I_L^{\alpha na}}{I_L^{\alpha na} + \kappa_{na}^{\alpha na}} - \xi - \delta_a \right) N_a \,, \tag{S5}$$

Germinal center B-cell dynamics:

$$\frac{dG_i}{dt} = \eta_i (1 - r_i) \xi N_a + \frac{\zeta_{mi} (1-q_i) \gamma_{mi} I_L^{\alpha m}}{I_L^{\alpha m} + \kappa_m^{\alpha m}} M_i + (2v_i - 1) \lambda_i G_i, \tag{S6}$$

where $v_i = \psi_i \Phi_i(I_G, G), \ \psi_i > 0$, and

$$\Phi_i(I_G, G) = \frac{I_G^{\alpha_g}}{I_G^{\alpha_g} + \kappa_{gi}^{\alpha_g}} \left[1 + \sum_{j=1}^{3} \delta_{ij} \, G_j(t) \right]^{-\Upsilon}, \quad i = 1, 2, 3, \ \ \delta_{ij} = 0, \ \ j < i. \tag{S7}$$

Short-lived antibody secreting cells dynamics:

$$\frac{dP_{si}}{dt} = u_i r_i \eta_i \xi N_a + \frac{\zeta_{mi} \, q_i \, \gamma_{mi} \, I_L^{\alpha m}}{I_L^{\alpha m} + \kappa_m^{\alpha m}} M_i + (1 - p_i) \lambda_i \varphi_i \, G_i - (w_i + \delta_s) \, P_{si} \,, \tag{S8}$$

Long-lived plasma B-cell dynamics:

$$\frac{dP_{li}}{dt} = w_i P_{si} - \delta_l P_{li} \,, \tag{S9}$$

Memory B-cell dynamics:

$$\frac{dM_i}{dt} = p_i \lambda_i \varphi_i G_i + (1 - u_i) r_i \, \eta_i \xi N_a - \left(\frac{\gamma_{mi} \, I_L^{\alpha m}}{I_L^{\alpha m} + \kappa_m^{\alpha m}} + \delta_m \right) M_i \,, \tag{S10}$$

Free antibody dynamics:

$$\frac{dA_i}{dt} = \sigma_s P_{si} + \sigma_l P_{Li} - \frac{\varrho \, \pi_i \, A_i^{\alpha_a}}{A_i^{\alpha_a} + \kappa_a^{\alpha_a}} I_L - c_A \, A_i \,, \quad i = 1, 2, 3. \tag{S11}$$

SM2. Model initial conditions

The ODE model in Eq. (S1) - (S11) describes the dynamics of B cells and antibodies induced by a protein-based immunogen and can be used to simulate both bolus and fractionated prime-boost vaccination regimens by specifying appropriate initial conditions. Here, we describe the initial conditions for both vaccination scenarios in the context of a clinical trial.

Vaccine doses in clinical trials are typically specified in terms of mass (grams); however, our model requires the number of immunogen molecules. Conversion from mass to molecule count is therefore necessary and is detailed in Section SM3. For the G001 trial consisting of two vaccine doses, at the injection site, the prime dose is specified as the initial condition $I_S(0) = I_{S1} > 0$. The

bioRxiv preprint doi: https://doi.org/10.64898/2026.10.14.759230; this version posted October 20, 2026. The copyright holder for this preprint (which was not certified by peer review) is the author/funder, who has granted bioRxiv a license to display the preprint in perpetuity. It is made available under a CC-BY 4.0 International license.

subsequent dose is given by $I_S(t_2) = I_{S2} > 0$, where $t_2 > 0$ is the administration time for the second dose. Since this is a homologous vaccine regimen, and the same amount (in grams) of vaccine was administered for both doses, we set $I_{S1} = I_{S2}$.

Since the model is applied to antigen-naïve individuals, i.e., those with no prior exposure to the immunogen, all immunogen and B-cell compartments are empty at the time of prime dose administration, except the injection site and vaccine-specific naïve B cells. Accordingly, the initial conditions for immunogens in the lymphatic compartments and GC are $I_L(0) = 0$ and $I_G(0) = 0$, respectively. For the naïve B cell population, we assume a positive constant initial population of vaccine-specific naïve B cells, $N(0) = N_0 > 0$. Since no vaccine-induced immune response exists prior to the prime dose, the initial populations of activated B cells, GC B cells, short-lived ASCs, long-lived plasma cells, and memory B cells are all set to zero, i.e., $N_a(0) = G_i(0) = P_{si}(0) = P_{li}(0) = M_i(0) = 0$, respectively. Similarly, there are no vaccine-induced antibodies at baseline, so $A_i(0) = 0$ for $i = 1, 2, 3$.

Putting these conditions together, we present the complete initial conditions for the model at the time of the prime dose:

$$\begin{gathered} I_S(0) = I_{S1} > 0, I_L(0) = 0, I_G(0) = 0, N(0) = N_0 > 0, N_a(0) = 0, \\ G_i(0) = P_{si}(0) = P_{li}(0) = M_i(0) = A_i(0) = 0, \qquad i = 1, \dots, 3. \end{gathered} \tag{S12}$$

SM3. Conversion of vaccine dose from mass to molecule count and antibody MFI to relative concentration

In the G001 clinical trial, vaccine doses were specified in terms of physical mass (micrograms, μg). However, our model tracks the number of immunogen molecules rather than mass. To convert the vaccine dose (in μg) to the number of immunogen molecules, we use the formula

$$N_{dose} = (V_{dose} \times C)/M_s,$$

where N_{dose} is the number of immunogen molecules in a vaccine dose V_{dose}, and M_s is the molecular mass of a single immunogen molecule (in Daltons), and $C = 6.024 \times 10^{17}$ Daltons/μg is the conversion factor from micrograms to Daltons, obtained from the relation $1\,\mu g = 6.024 \times 10^{17}$ Daltons. Since the eOD-GT8 60mer immunogen is a protein-based nanoparticle consisting of 60 identical subunits, each with a monomer mass of 67.7×10^3 Daltons, we set $M_s = 60 \times 67.7 \times 10^3 = 4.062 \times 10^6$ Daltons as the molecular mass of a single immunogen nanoparticle. Using this formula, we obtain $N_{dose} = 2.96 \times 10^{12}$ molecules for the 20 μg dose and $N_{dose} = 1.48 \times 10^{13}$ molecules for the 100 μg dose.

Furthermore, we estimated the relative antibody concentrations for each participant from the MFI measurement in the clinical trial data using a novel statistical method that integrates net MFI values across all available dilutions via quadratic B-splines with monotone constraints. The function describes the relationship between MFI and the log-transformed concentration of binding antibodies in each sample, with the monotone constraints ensuring a biologically consistent, non-decreasing relationship between the two quantities. The model was fitted to the data using the Newton-Raphson procedure, yielding estimates of relative antibody concentrations, i.e., unknown concentrations proportional to a common multiplicative factor shared across all samples. Antibody

bioRxiv preprint doi: https://doi.org/10.64898/2026.10.14.759230; this version posted October 20, 2026. The copyright holder for this preprint (which was not certified by peer review) is the author/funder, who has granted bioRxiv a license to display the preprint in perpetuity. It is made available under a CC-BY 4.0 International license.

concentration ratios were subsequently derived from these relative estimates. For example, the percentage of epitope-specific (P_S) antibodies were computed as:

$$P_S = \left(1 - \frac{R_K^c}{R_A^c}\right) \times 100\,,$$

where R_K^c and R_A^c refer to the relative concentration of vaccine-specific antibodies and the relative concentration of vaccine-specific antibodies binding to the mutated CD4bs KO immunogen, respectively. The calculations assumed that $C_A = C_K + C_S$, where C_A, C_K, and C_S refer to the concentration of antigen-specific, CD4bs-KO, and CD4bs-specific antibodies that bind to the CD4bs.

SM4. Model fit to the G001 data

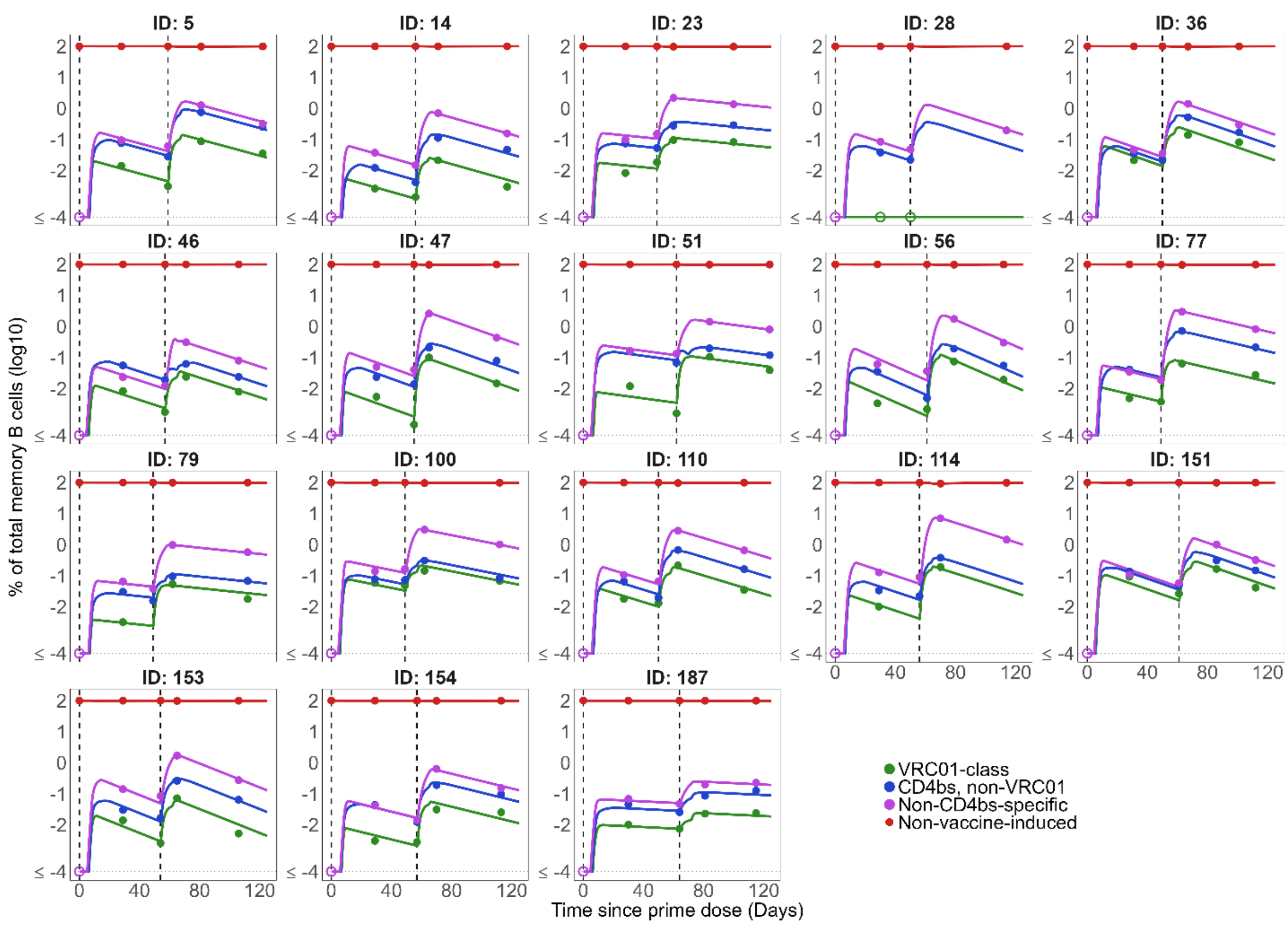

Figure SM1: Individual-level predictions for memory B-cell frequencies in the low-dose group. Observed (dots) and model-predicted (solid lines) frequencies of four memory B cell classes ($\log_{10}$) as a fraction of total memory B cells, for participants in the low-dose group of the G001 trial. The four classes are: VRC01-class (green), non-VRC01-class CD4bs-specific (blue), non-CD4bs-specific (purple), and non-vaccine-induced (red). Vertical dashed lines indicate vaccinations.

bioRxiv preprint doi: https://doi.org/10.64898/2026.10.14.759230; this version posted October 20, 2026. The copyright holder for this preprint (which was not certified by peer review) is the author/funder, who has granted bioRxiv a license to display the preprint in perpetuity. It is made available under a CC-BY 4.0 International license.

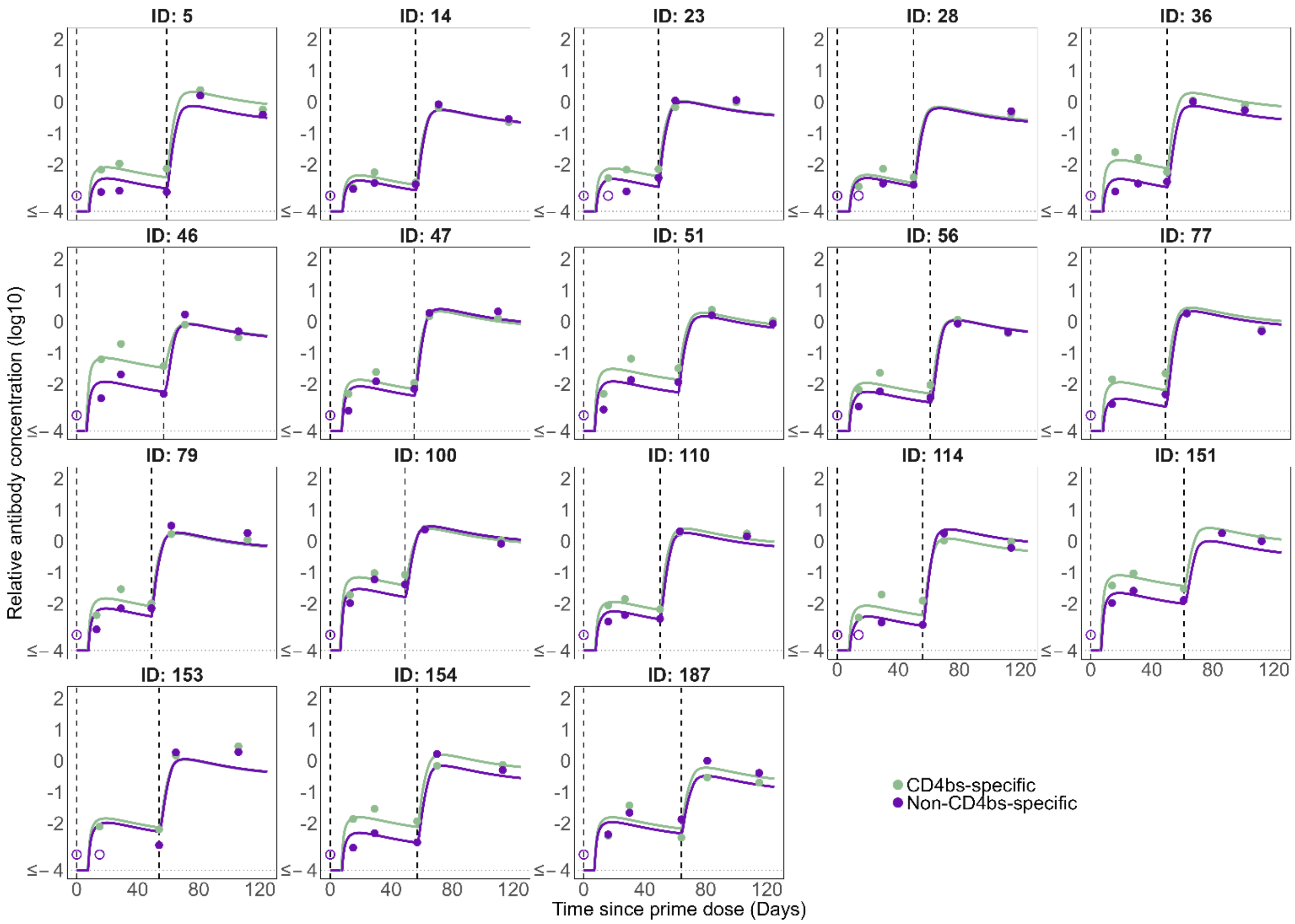

Figure SM2: Individual-level predictions for antibody levels in the low-dose group. Observed (dots) and model-predicted (solid lines) antibody levels ($\log_{10}$) for participants in the low-dose group of the G001 trial. Results are shown for two antibody specificities: CD4bs (purple) and non-CD4bs (green). Vertical dashed lines indicate vaccinations.

bioRxiv preprint doi: https://doi.org/10.64898/2026.10.14.759230; this version posted October 20, 2026. The copyright holder for this preprint (which was not certified by peer review) is the author/funder, who has granted bioRxiv a license to display the preprint in perpetuity. It is made available under a CC-BY 4.0 International license.

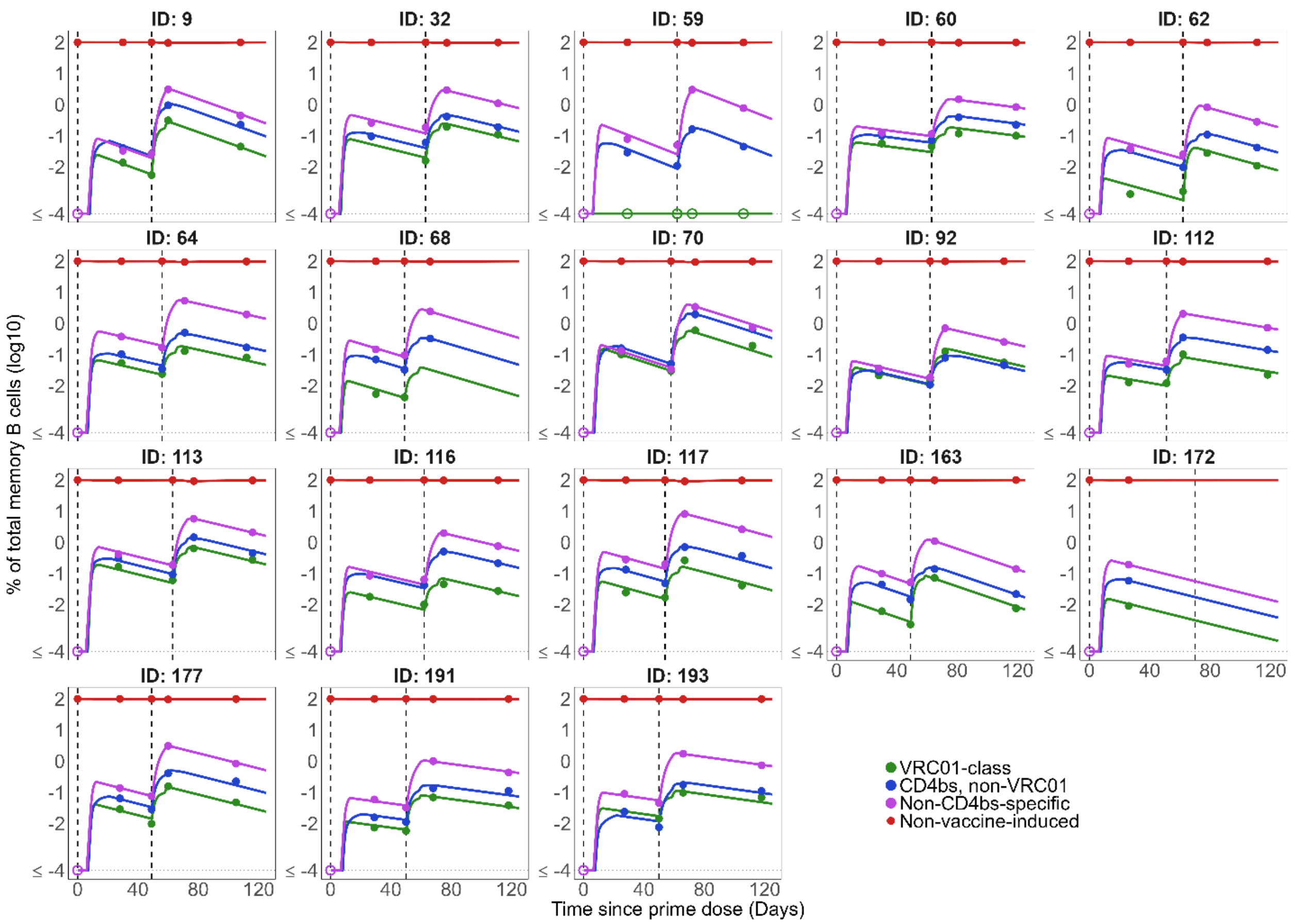

Figure SM3: Individual-level predictions for memory B-cell frequencies in the high-dose group. Observed (dots) and model-predicted (solid lines) frequencies of four memory B-cell classes ($\log_{10}$) as a fraction of total memory B cells, for participants in the high-dose group of the G001 trial. The four classes are: VRC01-class (green), non-VRC01-class CD4bs (blue), non-CD4bs (purple), and non-vaccine-induced (red). Vertical dashed lines indicate vaccinations.

bioRxiv preprint doi: https://doi.org/10.64898/2026.10.14.759230; this version posted October 20, 2026. The copyright holder for this preprint (which was not certified by peer review) is the author/funder, who has granted bioRxiv a license to display the preprint in perpetuity. It is made available under a CC-BY 4.0 International license.

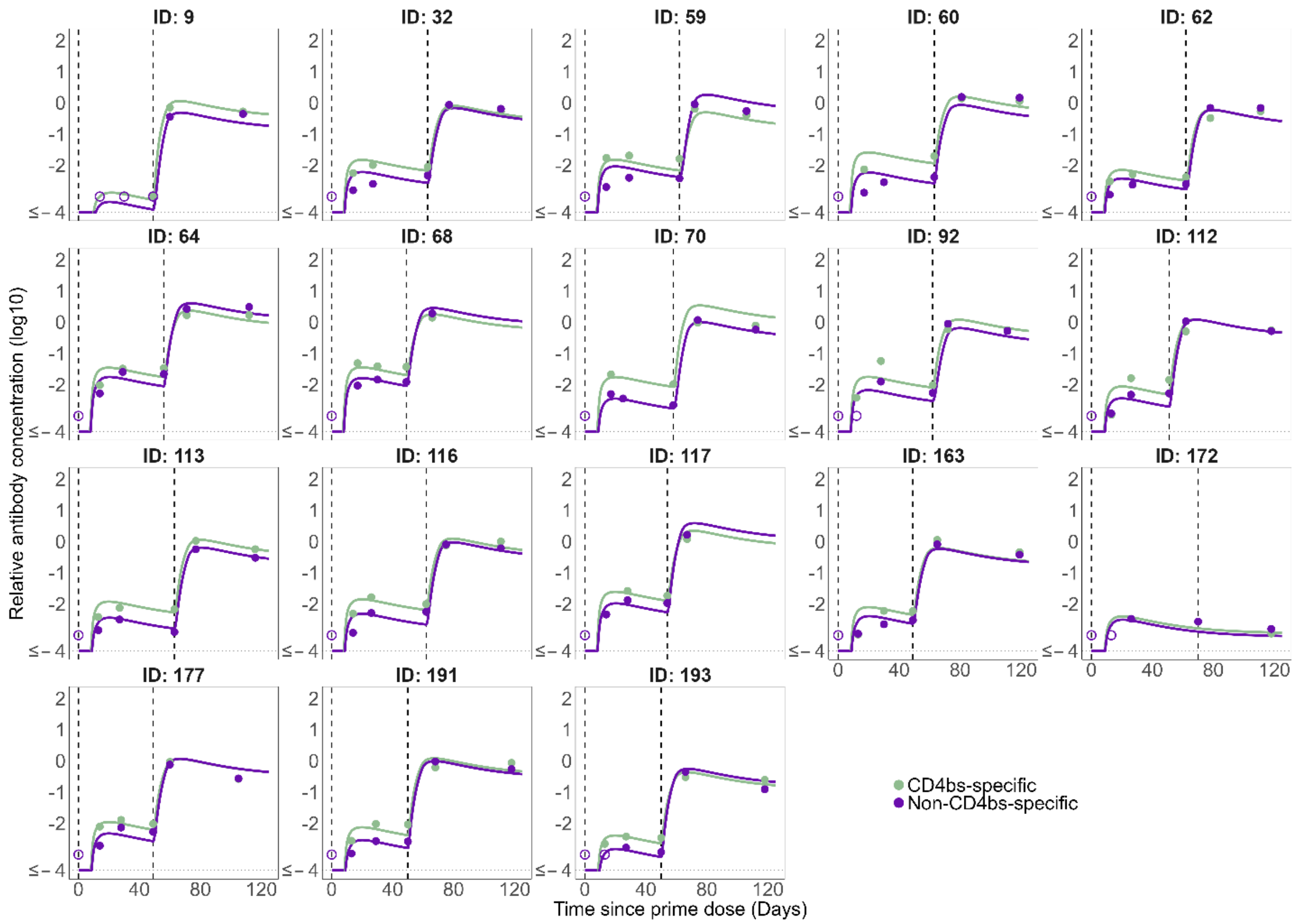

Figure SM4: Individual-level predictions for antibody levels in the high-dose group. Observed (dots) and model-predicted (solid lines) antibody levels ($\log_{10}$) for participants in the high-dose group of the G001 trial. Results are shown for two antibody specificities: CD4bs (gold) and non-CD4bs (green). Vertical dashed lines indicate vaccinations.

bioRxiv preprint doi: https://doi.org/10.64898/2026.10.14.759230; this version posted October 20, 2026. The copyright holder for this preprint (which was not certified by peer review) is the author/funder, who has granted bioRxiv a license to display the preprint in perpetuity. It is made available under a CC-BY 4.0 International license.

Table SM1: Model parameters and description

Parameter (unit)	Description	Value	Reference
ρ_s (/day)	Immunogen transfer rate from injection site to lymphatic compartments	2	Estimated
ρ_l (/day)	Immunogens transfer rate from lymphatic compartments to GC	2	Estimated
c_l (/day)	Immunogen degradation rate in the lymphatic compartments	1	Estimated
c_G (/day)	Immunogen degradation rate in the GC	1	Estimated
Λ_n (cells/day)	Production rate of vaccine-specific naïve B cells (N)	$\delta_n N_0$	Computed
γ_n (/day)	Activation rate of vaccine-specific naïve B cells by immunogens	See Table 2	Estimated
γ_{mi} (/day)	Activation rate of vaccine-induced memory B cells (M) of class i	See Table 2	Estimated
σ_{si} (Ab/Cell/day)	Antibody production rate by short-lived ASCs of class i	1.73×10^8	(1)
σ_{li} (Ab/Cell/day)	Antibody production rate by long-lived plasma cells of class i	1.73×10^8	(1)
π_i (/Ab/day)	Antibody-immunogen binding rate for antibodies of class i	1.0×10^{-7}	Fixed
c_A (/day)	Per capita clearance rate of free antibodies (A)	0.041	(2–4)
η_i	Fraction of activated-naïve B cells of class i	See Table 2	Estimated
r_i	Fraction of activated B cells of class i that differentiate into extrafollicular B cells	0.3	Estimated
u_i	Probability that an activated B cell of class i, upon differentiating along the extrafollicular pathway, becomes a short-lived ASC. $1 - u_i$ is the probability that the cell becomes a memory cell.	0.4	Estimated

bioRxiv preprint doi: https://doi.org/10.64898/2026.10.14.759230; this version posted October 20, 2026. The copyright holder for this preprint (which was not certified by peer review) is the author/funder, who has granted bioRxiv a license to display the preprint in perpetuity. It is made available under a CC-BY 4.0 International license.

$\boldsymbol{q_i}$	Fraction of activated memory B cells of class i that become short-lived ASCs (plasmablast)	0.9	Estimated
$\boldsymbol{p_i}$	Fraction of differentiated GC B cells of class i that become memory B cells	0.7	Estimated
$\boldsymbol{\xi}$ (/day)	Maturation rate of activated-naïve B cells before differentiating into extrafollicular B cells or entering the GC	0.45	Estimated
$\boldsymbol{\lambda}$ (/days)	Division/differentiation rate of GC B cells	4	(5–7)
$\boldsymbol{\psi}$	Conditional probability of division for a GC cell, given it did not die	0.5	Estimated
$\boldsymbol{\delta_n}$ (/day)	Per capita death rate of vaccine-specific naïve B cells	1/90	(8)
$\boldsymbol{\delta_a}$ (/day)	Per capita death rate of activated naïve B cells	1/3	(39)(39)
$\boldsymbol{\delta_s}$ (/day)	Per capita death rate of short-lived ASCs	1/5	(10–12)
$\boldsymbol{\delta_l}$ (/day)	Per capita death rate of long-lived plasma cells	See Table 2	Estimated
$\boldsymbol{\delta_m}$ (/day)	Per capita death rate of vaccine-induced memory B cells	See Table 2	Estimated
$\boldsymbol{\delta_{ij}}$ (/cell)	Suppression rate of GC B cell of class i by a GC B cell of class j	See Table 2	Estimated
$\boldsymbol{\zeta_a}$ (/day)	Proliferation rate of activated naïve B cells	2.5	Estimated
$\boldsymbol{\zeta_{mi}}$ (/day)	Average number of daughter cells per activated memory B cell of class i prior to differentiation	See Table 2	Estimated
$\boldsymbol{w}$ (/day)	Transition rate from short-lived ASCs to long-lived plasma cells	0.009	Estimated
$\boldsymbol{\alpha_n}$ ($\boldsymbol{\alpha_m}$)	Hill coefficient in the saturation function for the activation of naïve (memory) B cells	2	Fixed
$\boldsymbol{\alpha_a}$	Hill coefficient in the saturation function for antibody-immunogen binding, which captures the potential cooperativity in the binding interaction	2	Fixed
$\boldsymbol{\alpha_{na}}$	Hill coefficient in the saturation function for the proliferation of activated naïve B cells	2	Fixed

bioRxiv preprint doi: https://doi.org/10.64898/2026.10.14.759230; this version posted October 20, 2026. The copyright holder for this preprint (which was not certified by peer review) is the author/funder, who has granted bioRxiv a license to display the preprint in perpetuity. It is made available under a CC-BY 4.0 International license.

$\boldsymbol{\alpha_g}$	Hill coefficient in the saturation function used to modulate the effect of immunogens on the probability of division and differentiation of GC B cells	2	Fixed
$\boldsymbol{\kappa_n}$ ($\boldsymbol{\kappa_m}$) (Im)	Half-saturation constant for the saturation function used to modulate the effect of immunogens in the activation of naïve (memory) B cells	1.0×10^6	Fixed
$\boldsymbol{\kappa_{na}}$ (Im)	Half-saturation constant for the saturation function used to modulate the effect of immunogens in the proliferation of activated cells	1.0×10^6	Fixed
$\boldsymbol{\kappa_a}$ (Ab)	Half-saturation constant for the saturation function for antibody-immunogen binding, which modulates the sensitivity of the binding	1.0×10^{10}	Fixed
$\boldsymbol{\kappa_{gi}}$ (Im)	Half-saturation constant for the saturation function that modulates the effect of immunogens on the probability of cell division and differentiation in the GC for cells of class i	See Table 2	Estimated
$\boldsymbol{\tau}$ (Im/cell)	Scaling parameter representing the amount of immunogen required to activate naïve or memory B cells, or to drive proliferation of activated naïve B cells	1	Assumed
$\boldsymbol{\vartheta}$ (Im/cell)	Scaling parameter representing the amount of immunogen required for a GC B cell to undergo division or differentiation.	1	Assumed
$\boldsymbol{\varrho}$ (Im/cell)	Scaling parameter representing the stoichiometry of antibody–immunogen binding, i.e., the number of antibodies required per immunogen to form a stable complex.	1	Assumed
$\boldsymbol{\Upsilon}$	Controls the strength of the competition between GC B cells	0.5	Assumed
$\boldsymbol{\chi_{i,1}}$	Probability that a class-i GC B cell produces two class-i daughter cells upon division	1	Assumed
$\boldsymbol{\chi_{i,2}}$	Probability that a class-i GC B cell produces two class-$(i+1)$ daughter cells upon division	0	Assumed

bioRxiv preprint doi: https://doi.org/10.64898/2026.10.14.759230; this version posted October 20, 2026. The copyright holder for this preprint (which was not certified by peer review) is the author/funder, who has granted bioRxiv a license to display the preprint in perpetuity. It is made available under a CC-BY 4.0 International license.

$\chi_{i,3}$	Probability that a class-i GC B cell produces one class-i and one class-$(i+1)$ daughter cells upon division	0	Assumed
$\chi_{i,4}$	Probability that a class-i GC B cell produces two class-$(i-1)$ daughter cells upon division	0	Assumed
$\chi_{i,5}$	Probability that a class-i GC B cell produces one class-$(i-1)$ and one class-$(i+1)$ daughter cells upon division	0	Assumed
$\chi_{i,6}$	Probability that a class-i GC B cell produces one class-i and one class-$(i-1)$ daughter cells upon division	0	Assumed

SM5. Establishing the structural identifiability of GC B-cell pairwise competition coefficients

This section proves Theorem 1, which establishes the structural identifiability of the pairwise competition coefficients $\delta_{ij},\ i,j = 1,\dots,3$, between GC B-cell classes, when all other model parameters are fixed. The proof proceeds in two stages. The first stage reduces the uniqueness of the parameters $\delta = \left(\delta_{ij},\ i,j = 1,\dots,3\right)$ to a linear-independence property of the functions G_1, G_2, G_3. Specifically, by (S6), if the G001 Models parametrized by δ and $\delta' = \left(\delta'_{ij},\ i,j = 1,\dots,3\right)$ produce the same solution, then, for each i,

$$\sum_{j=1}^{3} \delta_{ij}\, G_j \ \equiv\ \sum_{j=1}^{3} \delta'_{ij}\, G_j, \qquad \text{(S13)}$$

on a neighborhood of 0^+. If the functions G_1, G_2, G_3 are linearly independent on this neighborhood, Eq. (S13) implies $\delta_{ij} = \delta'_{ij}$ for $j = 1, 2, 3$, and for every i, and hence $\delta = \delta'$. The second stage establishes this linear independence. We derive the local expansion of G_1, G_2, G_3 near $t = 0$ and show that no nontrivial linear combination of these expansions can vanish. This is the content of Theorem 2, which we prove first. Once Theorem 2 is established, the linear independence of G_1, G_2, G_3, together with (S13), yields $\delta = \delta'$. This completes the first stage of the argument and, consequently, the proof of Theorem 1. The remainder of this section is devoted first to Theorem 2 and then to returning to Theorem 1 at the end.

Linear independence of $G_1(t)$, $G_2(t)$, and $G_3(t)$ near $t = 0$

Let $t \in [0, T]$ for some $T \in (0, t_2)$, where t_2 denotes the time at which the second vaccination occurred. For any $\kappa, \alpha > 0$, let $H_{\kappa,\alpha}(x) := x^\alpha/(x^\alpha + \kappa^\alpha)$, and write $H_n := H_{\kappa_n,\alpha_n}$, $H_m := H_{\kappa_m,\alpha_m}$, $H_{na} := H_{\kappa_{na},\alpha_{na}}$, $H_a := H_{\kappa_a,\alpha_a}$, $H_{gi} := H_{\kappa_{gi},\alpha_g}$. At $t = 0$, we have that $I_S(0) = I_{S1} > 0$, $N(0) = N_0 := \Lambda_n/\delta_n$, and I_L, I_G, N_a, G_i, P_{si}, P_{li}, M_i, A_i are all 0. All rate constants, half-saturation constants, and Hill exponents are positive; $\eta_i, r_i, q_i, u_i, p_i, \varphi_i, \psi_i \in [0,1]$ with

bioRxiv preprint doi: https://doi.org/10.64898/2026.10.14.759230; this version posted October 20, 2026. The copyright holder for this preprint (which was not certified by peer review) is the author/funder, who has granted bioRxiv a license to display the preprint in perpetuity. It is made available under a CC-BY 4.0 International license.

$\eta_i(1-r_i) > 0$; $\delta_{ij} \geq 0$ and $\Upsilon > 0$. We assume that a continuous, nonnegative solution of (S1)-(S11) exists on $[0,T]$.

Define $a := \rho_{s1} I_{S1}$, $\mu := \min\{1, \alpha_n\}$, $n_0 := \frac{\gamma_n N_0}{\alpha_n+1}\left(\frac{a}{\kappa_n}\right)^{\alpha_n}$, and

$$\omega := \min\{2,\ 2\alpha_n,\ 1+\alpha_n,\ 1+\alpha_{na},\ 1+\alpha_m,\ 1+2\alpha_g,\ 1+\alpha_a(\alpha_n+3)\}.$$

In this analysis, the notations $O(\cdot)$ and $o(\cdot)$ refer to the limit $t \to 0^+$, with constants depending only on the model parameters and on T.

Theorem 2. *Assume* $\min(\alpha_m, 2\alpha_g) > 1$. *For* $k = 0,1,2$ *and* $t > 0$ *put* $J_k(t) := \frac{1}{k!}\int_0^t (t-s)^k\ N_a(s)\,ds$. *Then, for* $i = 1,2,3$,

$$G_i(t) = \eta_i(1-r_i)\,\xi[J_0(t) - \lambda_i J_1(t) + \lambda_i^2 J_2(t)] + o(t^{\alpha_n+4}), \qquad \text{as } t \to 0^+, \tag{S14}$$

where

$$J_k(t) = \beta_k\, t^{\alpha_n+2+k}\big(1+o(1)\big), \qquad \beta_k := \frac{n_0\,\Gamma(\alpha_n+2)}{\Gamma(\alpha_n+3+k)} > 0, \quad \text{as } t \to 0^+, \tag{S15}$$

with $\beta_0 = \frac{n_0}{\alpha_n+2}$, $\beta_1 = \frac{n_0}{(\alpha_n+2)(\alpha_n+3)}$, *and* $\beta_2 = \frac{n_0}{(\alpha_n+2)(\alpha_n+3)(\alpha_n+4)}$. *Consequently, if* $\lambda_1,\ \lambda_2,\ \lambda_3$ *are pairwise distinct, then* G_1, G_2, G_3 *are linearly independent on every interval* $(0,T)$.

Proof of Theorem 2

In this proof, we use four elementary facts, stated below.

F1. Let $X := (x/\kappa)^\alpha$. Then $H_{\kappa,\alpha}(x) = X - X^2/(1+X)$ and, when $x \geq 0$, $0 \leq X - H_{\kappa,\alpha}(x) \leq X^2$; in particular $H_{\kappa,\alpha}(x) = (x/\kappa)^\alpha + O((x/\kappa)^{2\alpha})$ and $H_{\kappa,\alpha}(x) \leq \min(X,1)$.

F2. $(1+\varepsilon)^\alpha = 1 + \alpha\varepsilon + O(\varepsilon^2)$ for $|\varepsilon| \leq \frac{1}{2}$.

F3. For an integer $k \geq 0$ and $\beta > -1$, substituting $s = tx$,

$$\int_0^t (t-s)^k\, s^\beta\, ds = \frac{k!\ \Gamma(\beta+1)}{\Gamma(\beta+k+2)}\ t^{\beta+k+1}.$$

F4. For $x \geq 0$, $0 \leq e^{-x} - (1-x) \leq \frac{x^2}{2}$ and $\left|e^{-x} - \left(1 - x + \frac{x^2}{2}\right)\right| \leq \frac{x^3}{6}$.

Step 1: preliminary bounds

We first develop bounds describing the behavior near $t = 0$ of certain functions appearing in the ODEs that define the G001 Model; these bounds will allow us to characterize the behavior of $G_i(t)$ near $t = 0$. To shorten notation, we use f' to denote the first derivative of any univariate function f. First, at any time $t \in [0,T]$, only the first immunization dose has been administered. Hence, by (S1), $I_S(t) = I_{S1}\, e^{-\rho_{s1} t}$. Dropping the nonpositive terms in (S2) and (S3) gives $I_L' \leq \rho_{s1} I_S \leq a$ and $I_G' \leq \rho_l I_L$, so that $I_L \leq at$ and $I_G \leq \frac{1}{2}\rho_l a\, t^2$. By (S4), $N' \leq \delta_n(N_0 - N)$, so $N \leq N_0$. Then

bioRxiv preprint doi: https://doi.org/10.64898/2026.10.14.759230; this version posted October 20, 2026. The copyright holder for this preprint (which was not certified by peer review) is the author/funder, who has granted bioRxiv a license to display the preprint in perpetuity. It is made available under a CC-BY 4.0 International license.

(S5) and F1 give $N_a{}' \leq \gamma_n N_0 (at/\kappa_n)^{\alpha_n} + \zeta_a N_a$. Integrating this inequality and using the fact that $N_a(0) = 0$, we obtain that $N_a = O(t^{\alpha_n+1})$. In (S6), (S8) and (S10), all coefficients are bounded ($H \leq 1$, $\Phi_i \leq 1$), so the derivative of $\sum_i (G_i + M_i + P_{si})$ is at most $c_0\,(N_a + \sum_i (G_i + M_i + P_{si}))$ for some constant $c_0 > 0$. Proceeding as done with N_a, we obtain that G_i, M_i, $P_{si} = O(t^{\alpha_n+2})$, and then (S9) and (S11) give $P_{li}, A_i = O(t^{\alpha_n+3})$. Therefore, every nonpositive term in (S2) is $O(t^\mu)$, and $\rho_{s1} I_S = a + O(t)$, such that $I_L{}' = a + O(t^\mu)$; hence $I_L = at\big(1 + O(t^\mu)\big)$. Finally, (S4) gives $N - N_0 = -\int_0^t e^{-\delta_n(t-s)}\, \gamma_n H_n(I_L) N\, ds = O(t^{\alpha_n+1})$, so $N = N_0\big(1 + o(1)\big)$.

Next, integrating (S2) from 0 to t and approximating each term with the above bounds gives

$$\int_0^t \rho_{s1}\, I_S\, ds = I_{S1}(1 - e^{-\rho_{s1} t}) = at - 1/2\, a\rho_{s1} t^2 + O(t^3),$$

and

$$(c_l + \rho_l) \int_0^t I_L\; ds = 1/2\,(c_l + \rho_l)\, a\, t^2 + O(t^{2+\mu}),$$

for the first and part of the second term, respectively. By F1 and F2, $H_n\big(I_L(s)\big) = (as/\kappa_n)^{\alpha_n}\big(1 + O(s^\mu)\big)$. Using $N = N_0 + O(s^{\alpha_n+1})$, the naïve B cell activation term gives

$$\tau \int_0^t \gamma_n\, H_n(I_L) N\, ds = \tau n_0\, t^{\alpha_n+1}\big(1 + O(t^\mu)\big).$$

Finally, the antibody, memory B cell, and activated B cell terms can be shown to be $O\big(t^{2+\alpha_a(\alpha_n+3)}\big)$, $O(t^{\alpha_n+\alpha_m+3})$, and $O(t^{\alpha_n+\alpha_{na}+2})$, respectively. After dividing by at, every term is $O(t^\omega)$. Hence,

$$I_L(t) = at\left[1 - \frac{\rho_{s1} + c_l + \rho_l}{2}\, t - \frac{\tau n_0}{a}\, t^{\alpha_n} + O(t^\omega)\right]. \tag{S16}$$

Step 2: Expansion of N_a near 0

Let $Q := \gamma_n H_n(I_L) N$. By Step 1, $(I_L/\kappa_n)^{\alpha_n} = (at/\kappa_n)^{\alpha_n}\big(1 + o(1)\big)$, which tends to 0 as $t \to 0^+$. Hence, F1 gives $H_n(I_L) = (at/\kappa_n)^{\alpha_n}\big(1 + o(1)\big)$. Since $N = N_0\big(1 + o(1)\big)$, we find that

$$Q(t) = (\alpha_n + 1)\, n_0\, t^{\alpha_n}\big(1 + o(1)\big).$$

Using (S5) and $N_a(0) = 0$,

$$N_a(t) = \int_0^t e^{-(\xi+\delta_a)(t-s)}\, [\zeta_a H_{na}(I_L) N_a(s) + Q(s)]\, ds.$$

In the first term, $H_{na}(I_L) = O(t^{\alpha_{na}})$ by F1 and $N_a(s) = O(s^{\alpha_n+1})$ by Step 1. Therefore, $\zeta_a H_{na}(I_L) N_a(s) = O(s^{\alpha_n+\alpha_{na}+1})$. Since $e^{-(\xi+\delta_a)(t-s)} \leq 1$ for $s \in [0, t]$, integrating this term over $[0, t]$ increases the order to $O(t^{\alpha_n+\alpha_{na}+2})$, which is $o(t^{\alpha_n+1})$.

For the second term, expand the kernel by one order. Put $c := \xi + \delta_a > 0$. By the first bound in F4 with $x = c(t - s) \in [0, ct]$,

bioRxiv preprint doi: https://doi.org/10.64898/2026.10.14.759230; this version posted October 20, 2026. The copyright holder for this preprint (which was not certified by peer review) is the author/funder, who has granted bioRxiv a license to display the preprint in perpetuity. It is made available under a CC-BY 4.0 International license.

$$e^{-c(t-s)} = 1 - c(t-s) + R(t,s), \qquad |R(t,s)| \le \frac{c^2}{2}(t-s)^2 \le \frac{c^2}{2}\,t^2,$$

so that

$$\int_0^t e^{-c(t-s)}\,Q(s)\,ds = \int_0^t Q\,(s)\,ds \;-\; c\int_0^t (t-s)\;Q(s)\,ds \;+\; O(t^2)\int_0^t Q\,(s)\,ds.$$

Write $Q(s) = (\alpha_n + 1)\,n_0\,s^{\alpha_n}\big(1 + \varepsilon(s)\big)$ with $\varepsilon(s) \to 0$ as $s \to 0^+$. By F3 with $\beta = \alpha_n$,

$$\int_0^t s^{\alpha_n}\,ds = \frac{t^{\alpha_n+1}}{\alpha_n+1}, \quad (k=0); \qquad \int_0^t (t-s)\;s^{\alpha_n}\,ds = \frac{t^{\alpha_n+2}}{(\alpha_n+1)(\alpha_n+2)}, \quad (k=1),$$

and in each case the ε-part is bounded by $\big(\sup_{[0,t]}|\varepsilon|\big)$ times the same quantity. The three terms above are therefore

$$n_0\,t^{\alpha_n+1}\big(1+o(1)\big), \qquad \frac{c\,n_0}{\alpha_n+2}\,t^{\alpha_n+2}\big(1+o(1)\big), \quad \text{and} \quad O(t^{\alpha_n+3}).$$

The last two are $o(t^{\alpha_n+1})$, as is the ζ_a contribution. Hence, we can only retain the first, and

$$N_a(t) = n_0\,t^{\alpha_n+1}\big(1+o(1)\big). \tag{S17}$$

Step 3: Expansion of G_i near 0

Since $v_i = \psi_i\Phi_i$, (S6) can be expressed as

$$G_i' = -\lambda_i G_i + \eta_i(1-r_i)\,\xi\,N_a + [\zeta_{mi}(1-q_i)\gamma_{mi}H_m(I_L)M_i + 2\psi_i\lambda_i\Phi_i G_i]. \tag{S18}$$

Using F1 and Step 1 gives $H_m(I_L) = O(t^{\alpha_m})$ and $\Phi_i \le H_{gi}(I_G) = O(t^{2\alpha_g})$, while $M_i, G_i = O(t^{\alpha_n+2})$ by Step 1. Hence, the order of the term in the bracket is $O\big(t^{\alpha_n+2+\min(\alpha_m,2\alpha_g)}\big)$. Since $G_i(0) = 0$, integrating (S18) gives

$$G_i(t) = \eta_i(1-r_i)\,\xi\int_0^t e^{-\lambda_i(t-s)}\,N_a(s)\,ds + O\big(t^{\alpha_n+3+\min(\alpha_m,2\alpha_g)}\big). \tag{S19}$$

Using the Taylor expanding of $e^{-\lambda_i(t-s)}$ as $\mathrm{t} \to 0^+$ in the integral in $G_i(t)$ together with $N_a = O(s^{\alpha_n+1})$, we obtain

$$\int_0^t \left(1 - \lambda_i(t-s) + \frac{\lambda_i^2}{2}(t-s)^2 + \frac{\lambda_i^3}{6}(t-s)^3 + O(t-s)^4\right) N_a(s)\,ds$$
$$= J_0 - \lambda_i J_1 + \lambda_i^2 J_2 + O(t^{\alpha_n+5}),$$

where $J_k(t) := \frac{1}{k!}\int_0^t (t-s)^k\;N_a(s)\,\mathrm{d}s$. Substituting this result back into (S19), we obtain

$$G_i(t) = \eta_i(1-r_i)\,\xi[J_0(t) - \lambda_i J_1(t) + \lambda_i^2 J_2(t)] + O\big(t^{\alpha_n+3+\min(\alpha_m,2\alpha_g)}\big) + O(t^{\alpha_n+5}).$$

Because $\min(\alpha_m, 2\alpha_g) > 1$, both error terms are $o(t^{\alpha_n+4})$, which gives $G_i(t)$ as presented in (S14). Finally, inserting (S17) into the definition of J_k and applying F3 with $\beta = \alpha_n + 1$ gives (S15); the stated values of $\beta_0, \beta_1, \beta_2$ follow from $\Gamma(z+1) = z\Gamma(z)$.

bioRxiv preprint doi: https://doi.org/10.64898/2026.10.14.759230; this version posted October 20, 2026. The copyright holder for this preprint (which was not certified by peer review) is the author/funder, who has granted bioRxiv a license to display the preprint in perpetuity. It is made available under a CC-BY 4.0 International license.

Step 4: Linear independence of G_1, G_2, and G_3

Assume that λ_1, λ_2, and λ_3 are pairwise distinct, suppose $\sum_i c_i\, G_i \equiv 0$ on some $(0, \hat{T})$, and set $x_i := c_i\, \eta_i(1 - r_i)$. Summing (S14) against the c_i yields

$$\xi\left[\left(\sum_{i=1}^{3} x_i\right)J_0(t) - \left(\sum_{i=1}^{3} x_i\,\lambda_i\right)J_1(t) + \left(\sum_{i=1}^{3} x_i\,\lambda_i^2\right)J_2(t)\right] = o(t^{\alpha_n+4}). \qquad \text{(S20)}$$

We extract the three sums in turn, using (S15). Divide (S20) by t^{α_n+2} and let $t \to 0^+$. By (S15), $J_0/t^{\alpha_n+2} \to \beta_0 > 0$, while $J_1/t^{\alpha_n+2} \to 0$ and $J_2/t^{\alpha_n+2} \to 0$, and the right-hand side tends to 0. Hence $\sum_i x_i = 0$. With $\sum_i x_i = 0$ the first term of (S20) vanishes identically, not merely to leading order. Divide what remains by t^{α_n+3} and let $t \to 0^+$: now $J_1/t^{\alpha_n+3} \to \beta_1 > 0$ and $J_2/t^{\alpha_n+3} \to 0$, giving $\sum_i x_i\,\lambda_i = 0$. This makes the second term vanish. Dividing the remainder by t^{α_n+4} and letting $t \to 0^+$, with $J_2/t^{\alpha_n+4} \to \beta_2 > 0$, gives $\sum_i x_i\,\lambda_i^2 = 0$. Thus, x_1, x_2, x_3 solve the linear system

$$\begin{pmatrix} 1 & 1 & 1 \\ \lambda_1 & \lambda_2 & \lambda_3 \\ \lambda_1^2 & \lambda_2^2 & \lambda_3^2 \end{pmatrix}\begin{pmatrix} x_1 \\ x_2 \\ x_3 \end{pmatrix} = \begin{pmatrix} 0 \\ 0 \\ 0 \end{pmatrix}.$$

The determinant of the Vandermonde matrix is $\prod_{i<j}(\lambda_j - \lambda_i) \neq 0$ because the λ_i are pairwise distinct. The system therefore has only the solution $x_1 = x_2 = x_3 = 0$, and since $\eta_i(1 - r_i) > 0$ we get $c_i = x_i/\eta_i(1 - r_i) = 0$ for every i. This completes the proof of Theorem 2.

Proof of Theorem 1

If $\delta = \delta'$ the two models coincide. Suppose now that the models parametrized by δ and δ' produce the same solution of (S1)-(S11) on $[0, T]$, and write Φ_i and Φ'_i for the two versions of the competition factor,

$$\Phi_i = H_{gi}(I_G)\left(1 + \sum_{j=1}^{3} \delta_{ij}\, G_j\right)^{-\Upsilon}, \qquad \Phi'_i = H_{gi}(I_G)\left(1 + \sum_{j=1}^{3} \delta'_{ij}\, G_j\right)^{-\Upsilon}.$$

Fix i. Every term of (S6) other than $2\psi_i\lambda_i\Phi_i G_i$ is identical under the two parametrizations, as is G_i'; hence $2\psi_i\lambda_i\Phi_i G_i = 2\psi_i\lambda_i\Phi'_i G_i$ on $[0, T]$. Since $\psi_i > 0$ and $\lambda_i > 0$, this gives $\Phi_i G_i = \Phi'_i G_i$.

By (S14) and (S15), $G_i(t) = \eta_i(1 - r_i)\,\xi\,\beta_0\, t^{\alpha_n+2}(1 + o(1))$ with $\eta_i(1 - r_i)\,\xi\,\beta_0 > 0$, so there is $T^* \in (0, T]$ with $G_i(t) > 0$ for all $t \in (0, T^*)$. On that interval, we may cancel G_i, so $\Phi_i = \Phi'_i$. Moreover $I_G' = \rho_l I_L - \theta \sum_j \lambda_j\, v_j G_j - c_G I_G$, and by Step 1 the first term is $\rho_l a t(1 + o(1))$ while the others are $O(t^{\alpha_n+2})$ and $O(t^2)$; hence $I_G(t) > 0$, and therefore $H_{gi}(I_G) > 0$, after shrinking T^* if necessary. Dividing by $H_{gi}(I_G)$ leaves

$$\left(1 + \sum_{j=1}^{3} \delta_{ij}\, G_j\right)^{-\Upsilon} = \left(1 + \sum_{j=1}^{3} \delta'_{ij}\, G_j\right)^{-\Upsilon} \qquad \text{on } (0, T^*).$$

bioRxiv preprint doi: https://doi.org/10.64898/2026.10.14.759230; this version posted October 20, 2026. The copyright holder for this preprint (which was not certified by peer review) is the author/funder, who has granted bioRxiv a license to display the preprint in perpetuity. It is made available under a CC-BY 4.0 International license.

Both bases are at least 1 because $\delta_{ij}, \delta'_{ij} \geq 0$ and $G_j \geq 0$, and $u \mapsto u^{-\Upsilon}$ is strictly decreasing on $(0, \infty)$ since $\Upsilon > 0$; the bases therefore coincide, which is (S13), that is,

$$\sum_{j=1}^{3} \left(\delta_{ij} - \delta'_{ij}\right) G_j \equiv 0 \qquad \text{on } (0, T^*).$$

By Theorem 2, G_1, G_2, G_3 are linearly independent on $(0, T^*)$, so $\delta_{ij} = \delta'_{ij}$ for $j = 1, 2, 3$. As i was arbitrary, $\delta = \delta'$. This completes the proof of Theorem 1. ■

SM6. Profile likelihood for the GC B-cell competition coefficients

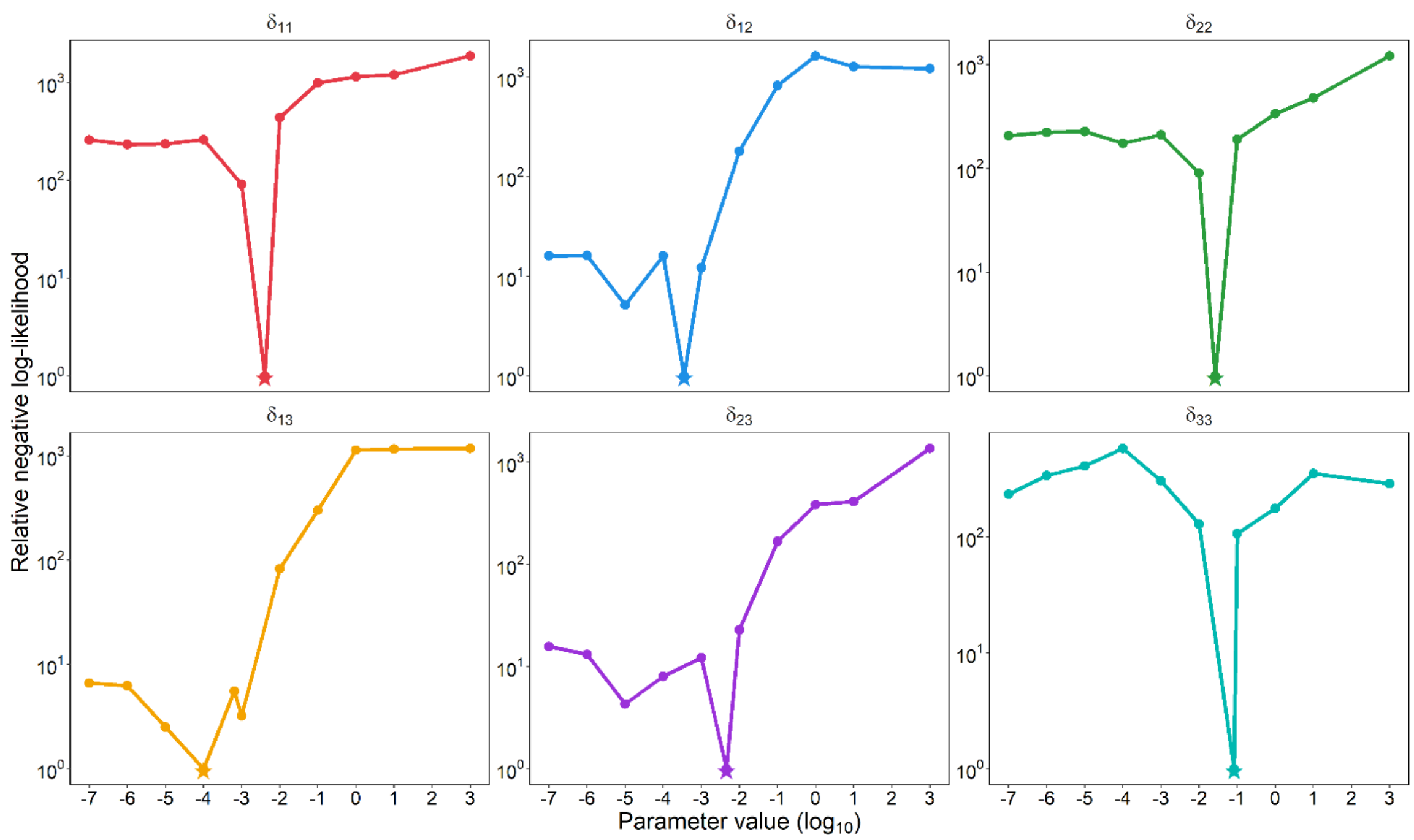

Figure SM5. Profile-likelihood results for the germinal center B-cell competition coefficients. Each panel shows the negative log-likelihood (NLL) for each GC B-cell competition coefficient ($\log_{10}$ scale) at different values of the parameter, relative to the minimum NLL over the entire parameter set (offset by 1 for display on a logarithmic scale). The star marks the estimated value of each competition coefficient reported in Table 2.

bioRxiv preprint doi: https://doi.org/10.64898/2026.10.14.759230; this version posted October 20, 2026. The copyright holder for this preprint (which was not certified by peer review) is the author/funder, who has granted bioRxiv a license to display the preprint in perpetuity. It is made available under a CC-BY 4.0 International license.

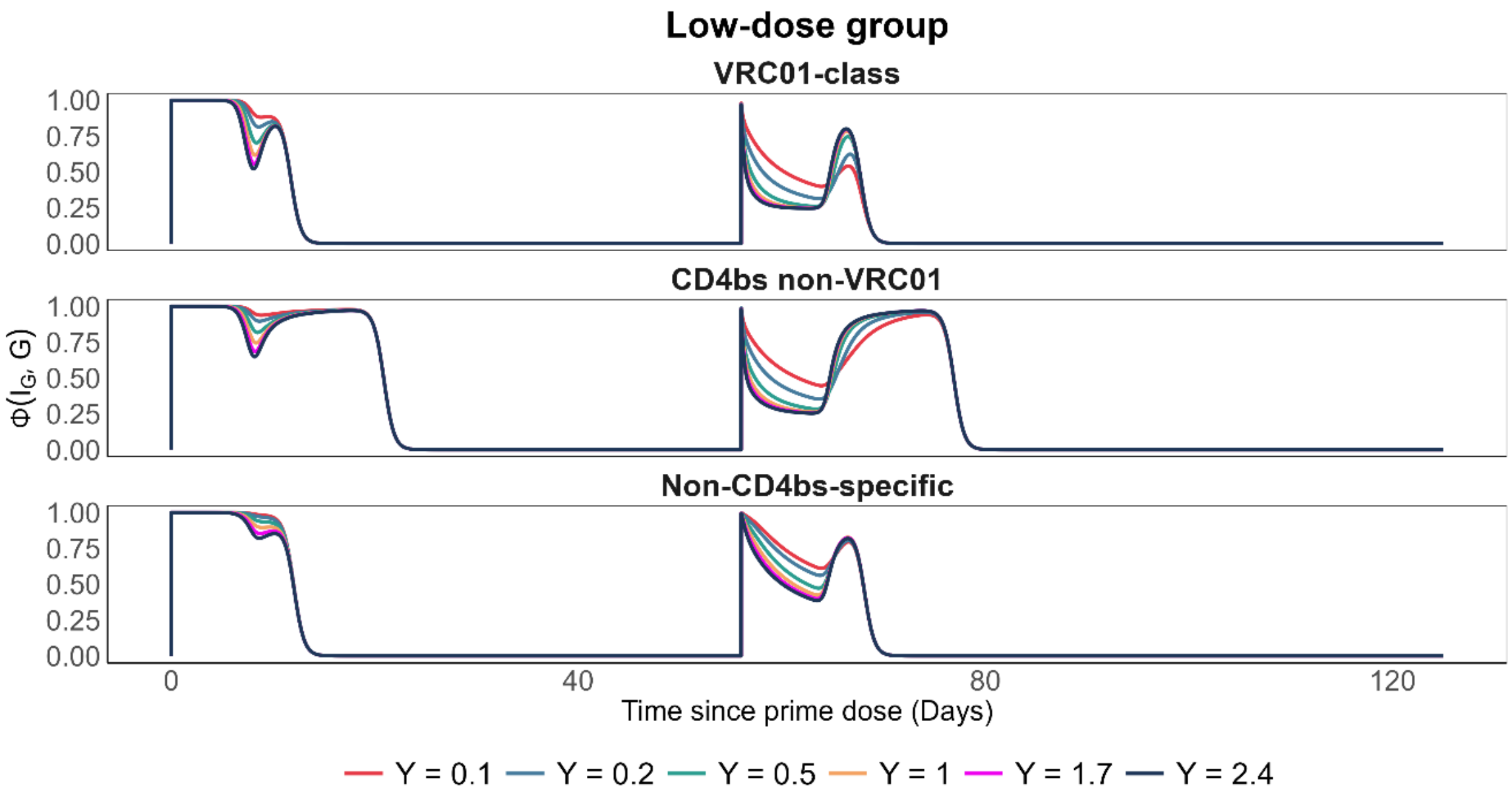

Figure SM6. Simulated trajectories for the probability of division or differentiation (low-dose group). Probability of division or differentiation $\Phi(I_G, G)$ over time (days) for the three B-cell classes (VRC01-class, CD4bs non-VRC01, and non-CD4bs-specific) in the low-dose group, shown for six representative values of ϒ.

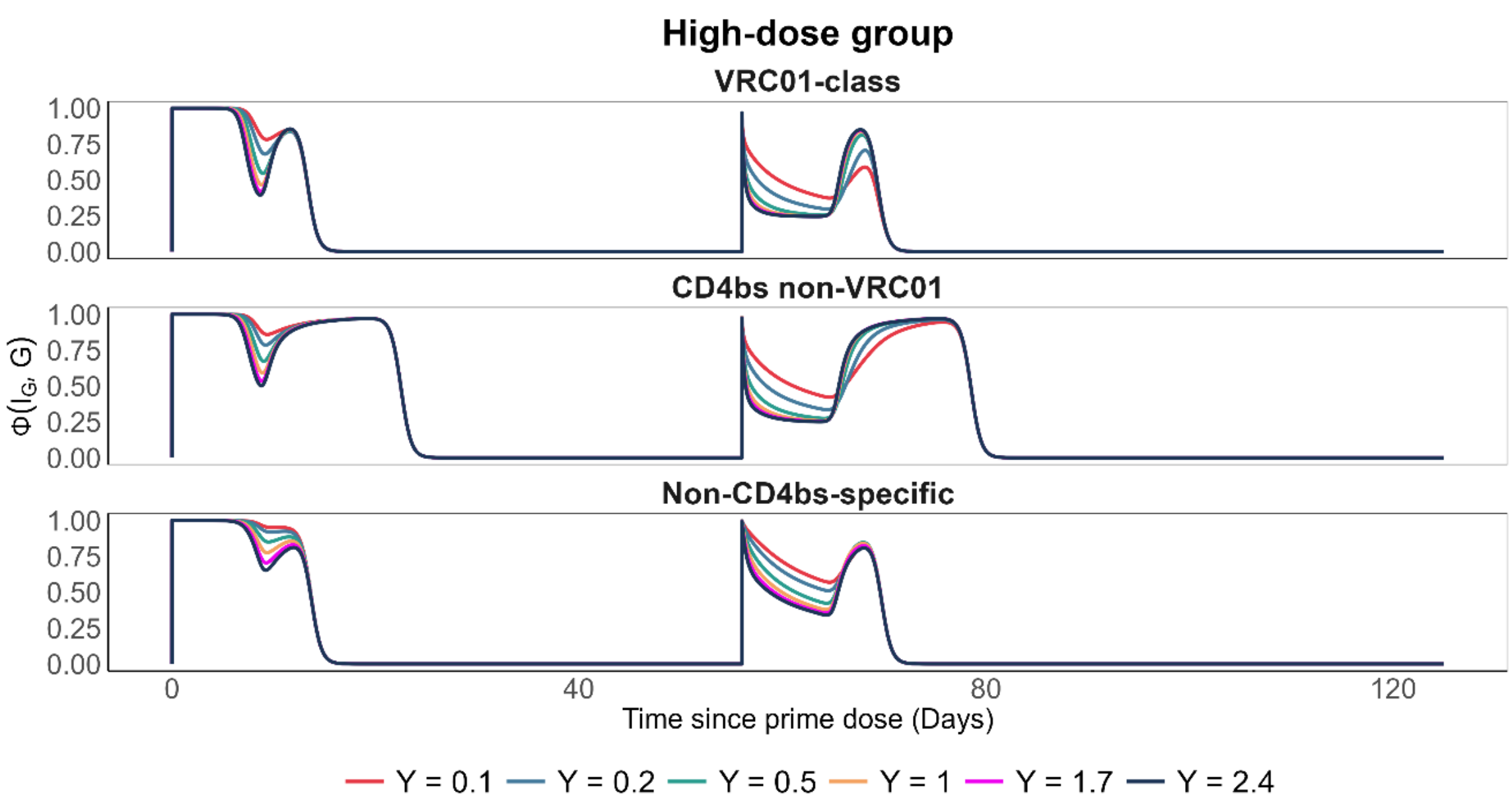

Figure SM7. Simulated trajectories for the probability of division or differentiation (high-dose group). Probability of division or differentiation $\Phi(I_G, G)$ over time (days) for the three B-cell classes (VRC01-class, CD4bs non-VRC01, and non-CD4bs-specific) in the low-dose group, shown for six representative values of ϒ.

bioRxiv preprint doi: https://doi.org/10.64898/2026.10.14.759230; this version posted October 20, 2026. The copyright holder for this preprint (which was not certified by peer review) is the author/funder, who has granted bioRxiv a license to display the preprint in perpetuity. It is made available under a CC-BY 4.0 International license.

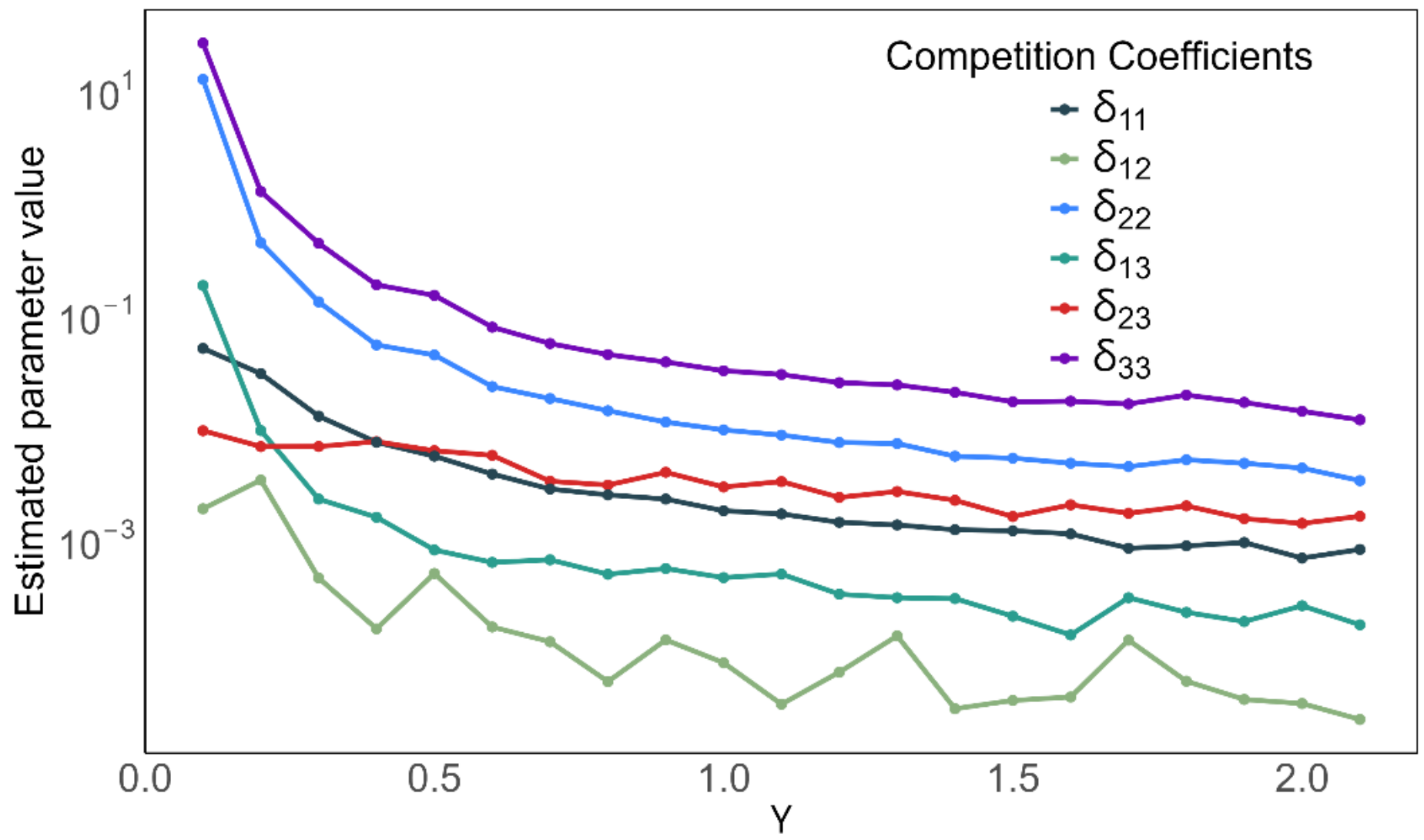

Figure SM8. Estimated GC B cells competition coefficients as a function of ϒ.

bioRxiv preprint doi: https://doi.org/10.64898/2026.10.14.759230; this version posted October 20, 2026. The copyright holder for this preprint (which was not certified by peer review) is the author/funder, who has granted bioRxiv a license to display the preprint in perpetuity. It is made available under a CC-BY 4.0 International license.

SM7. Effect of antibody masking on immune response dynamics

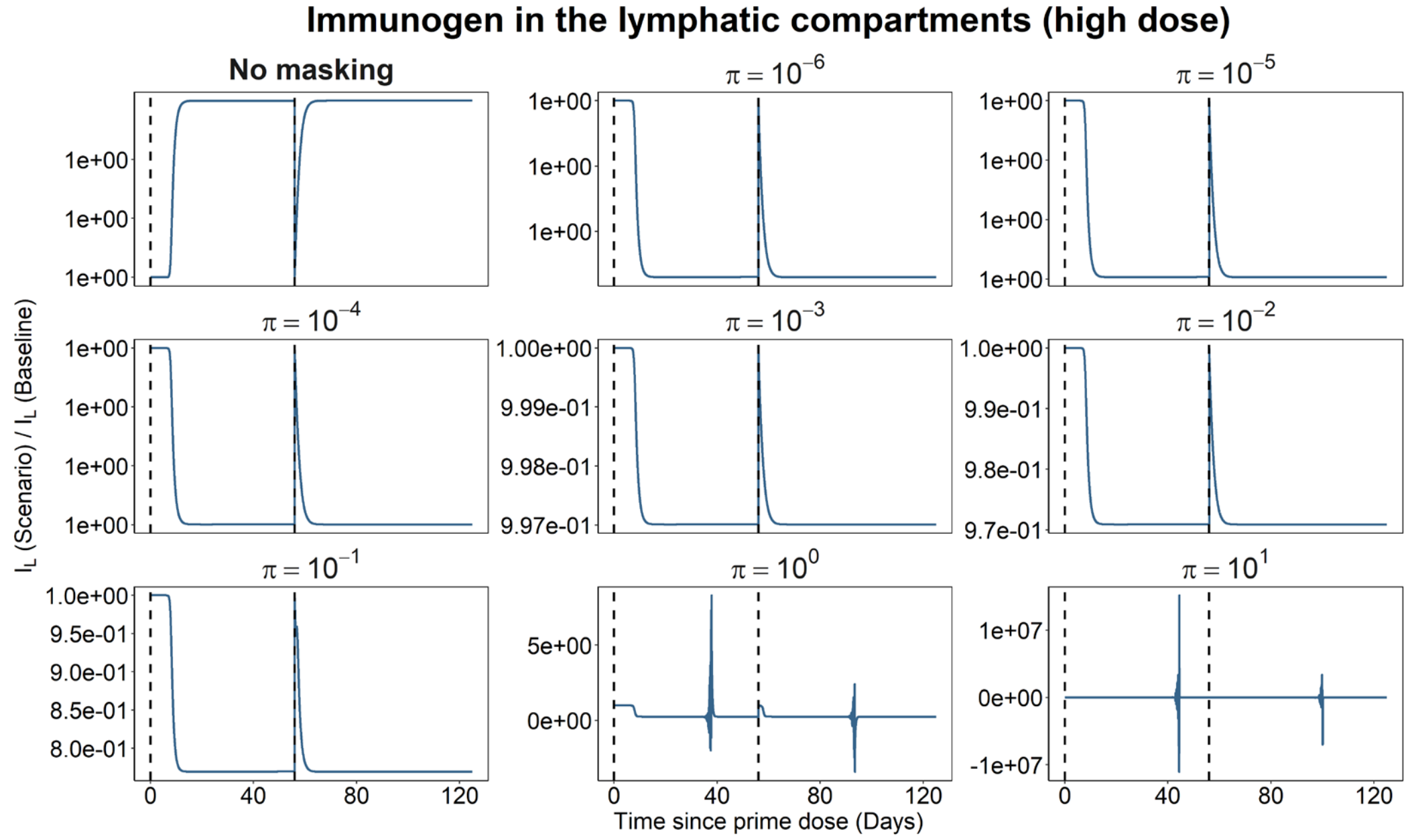

Figure SM9: Effect of antibody masking on immunogen dynamics in the lymphatic compartment. Ratio of immunogen levels in the lymphatic compartment for different values of the immunogen–antibody binding rate (π) relative to the immunogen level at $\pi = 10^{-7}$ (baseline).

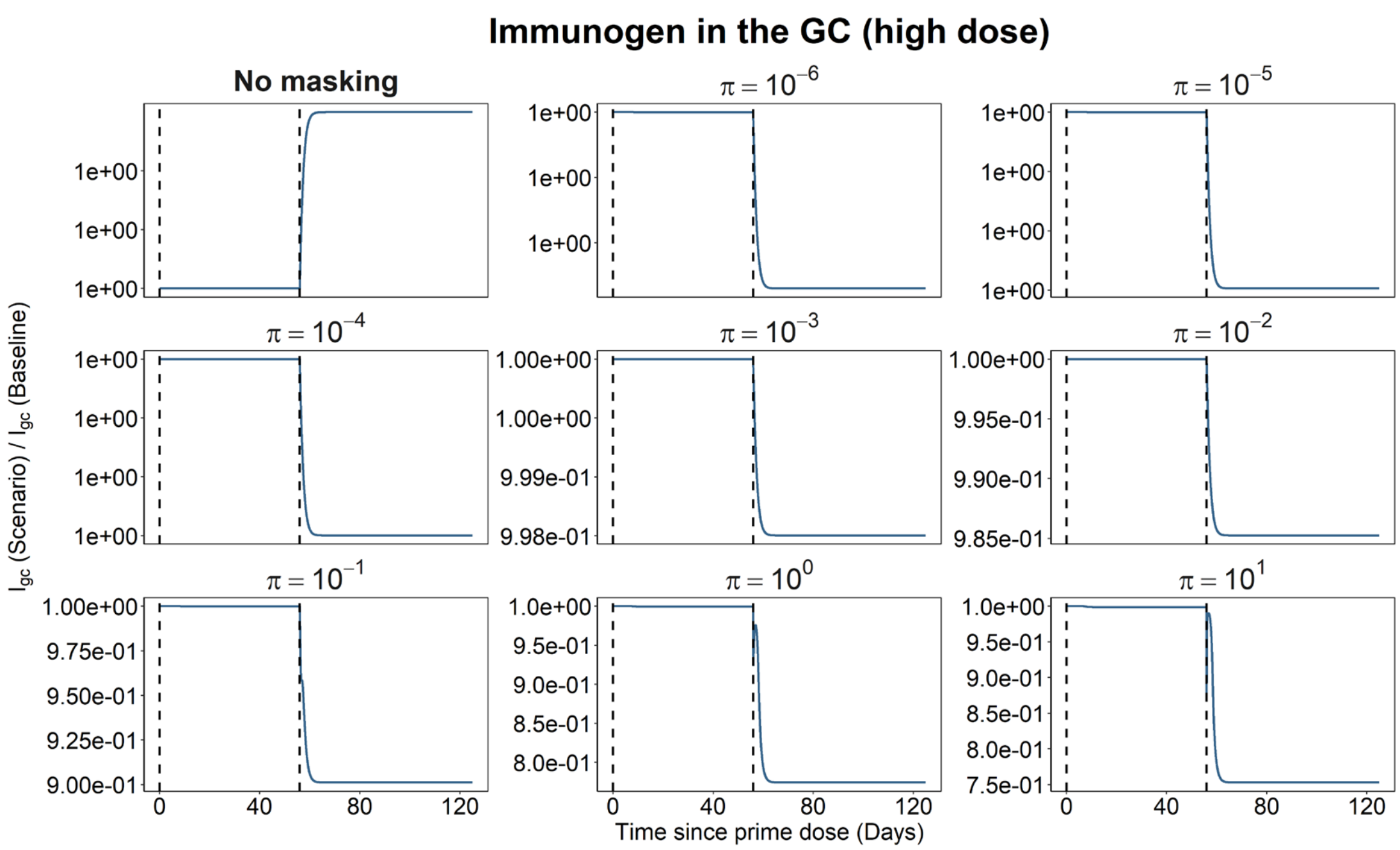

Figure SM10: Effect of antibody masking on immunogen dynamics in the GC. Ratio of immunogen levels in the GC for different values of the immunogen–antibody binding rate (π) relative to the immunogen level at $\pi = 10^{-7}$ (baseline).

bioRxiv preprint doi: https://doi.org/10.64898/2026.10.14.759230; this version posted October 20, 2026. The copyright holder for this preprint (which was not certified by peer review) is the author/funder, who has granted bioRxiv a license to display the preprint in perpetuity. It is made available under a CC-BY 4.0 International license.

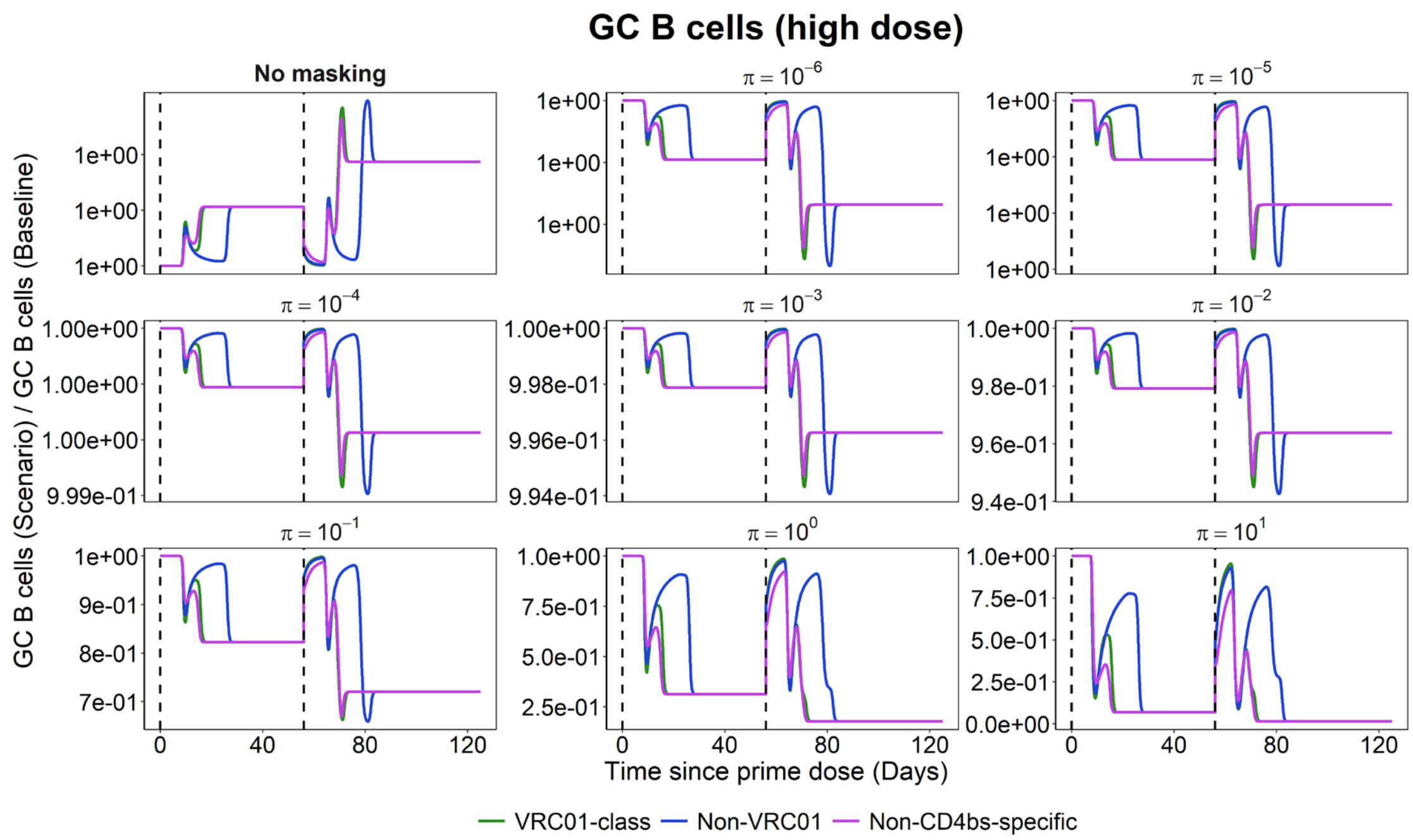

Figure SM11: Effect of antibody masking on vaccine-induced GC B-cell dynamics. Ratio of GC B cells for different values of the immunogen–antibody binding rate (π) relative to the GC B cells at $\pi = 10^{-7}$ (baseline).

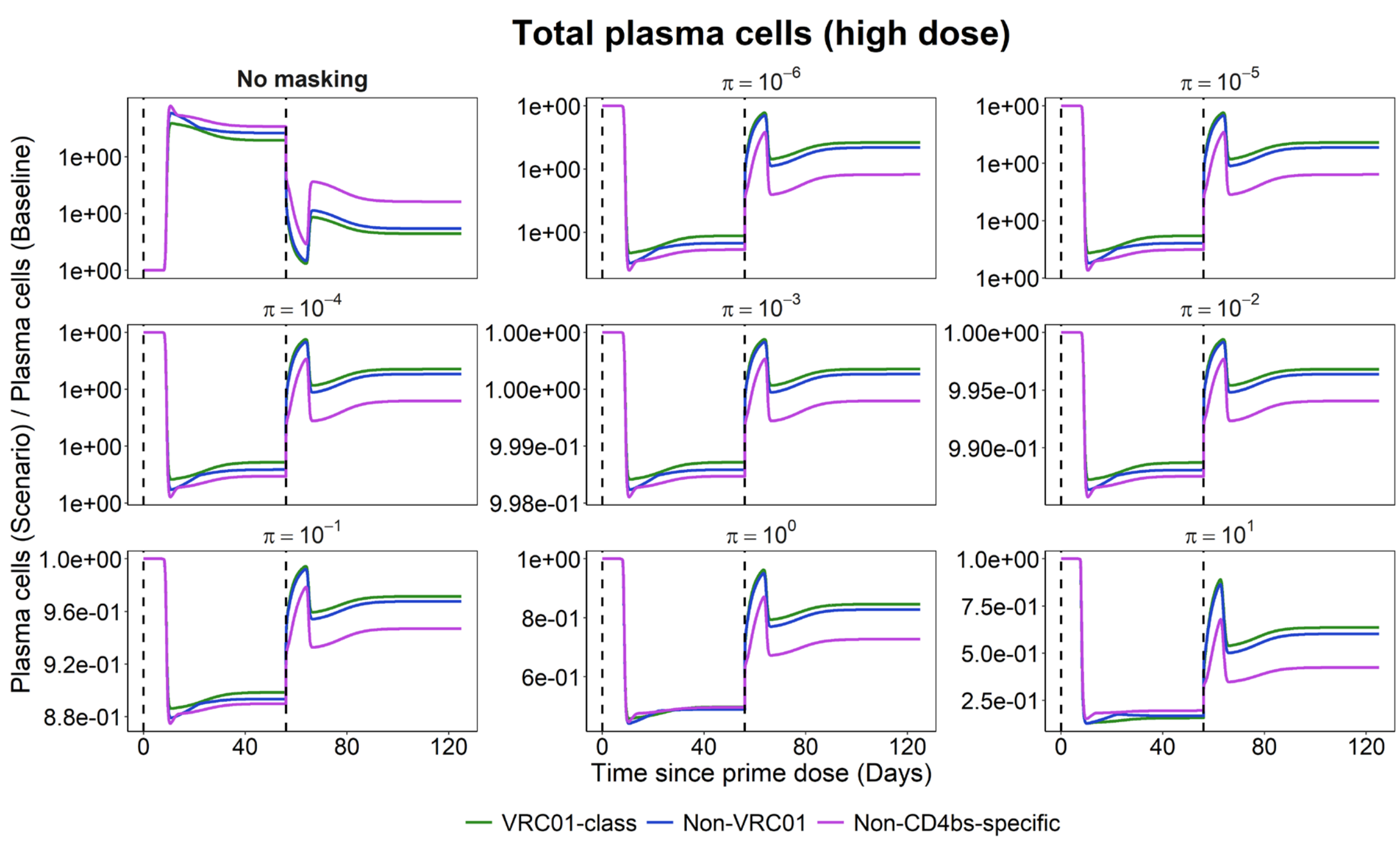

Figure SM12: Effect of antibody masking on vaccine-induced plasma-cell dynamics. Ratio of plasma cells for different values of the immunogen–antibody binding rate (π) relative to the plasma cells at $\pi = 10^{-7}$ (baseline).

bioRxiv preprint doi: https://doi.org/10.64898/2026.10.14.759230; this version posted October 20, 2026. The copyright holder for this preprint (which was not certified by peer review) is the author/funder, who has granted bioRxiv a license to display the preprint in perpetuity. It is made available under a CC-BY 4.0 International license.

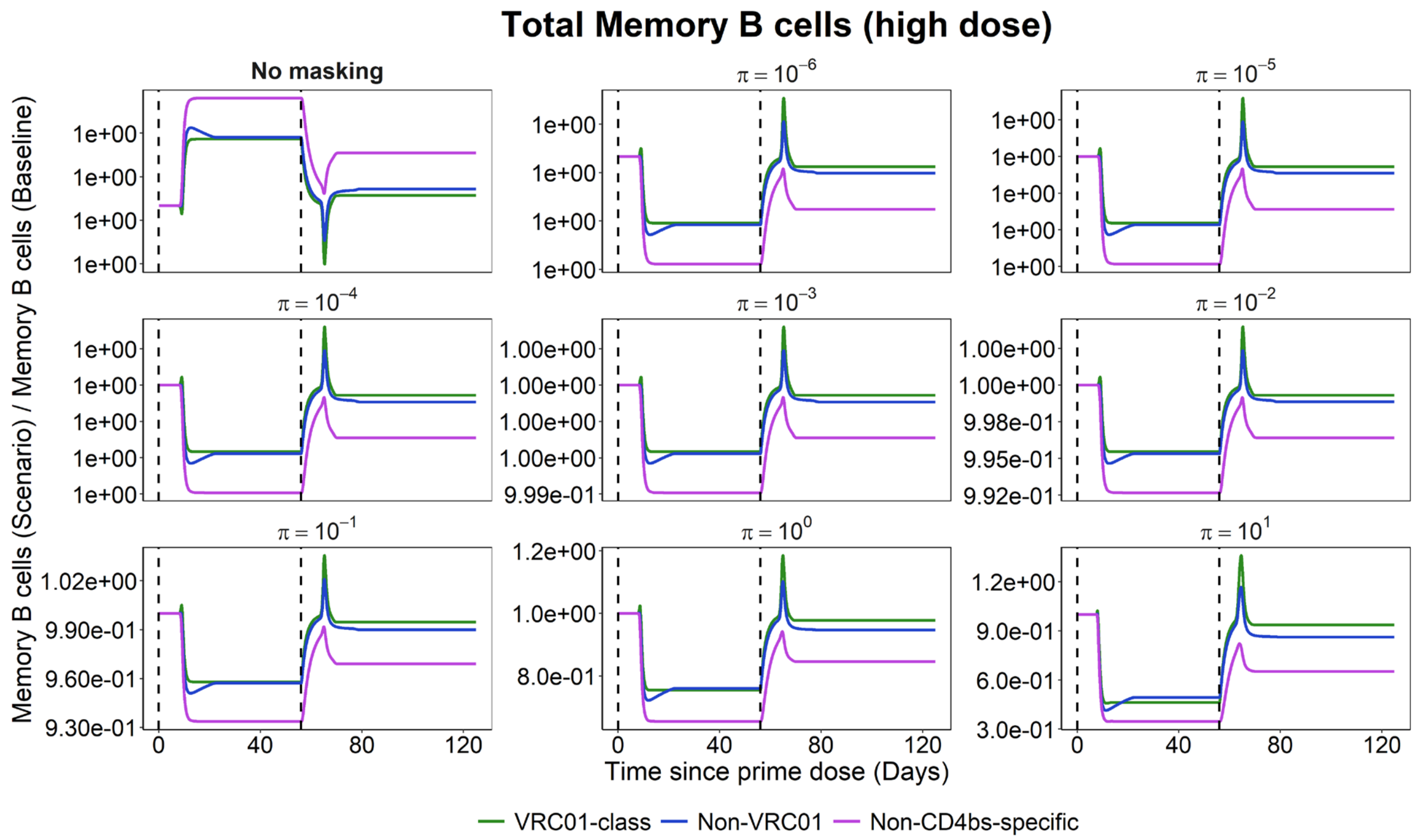

Figure SM13: Effect of antibody masking on vaccine-induced memory B-cell dynamics. Ratio of memory B cells for different values of the immunogen–antibody binding rate (π) relative to the memory B cells at $\pi = 10^{-7}$ (baseline).

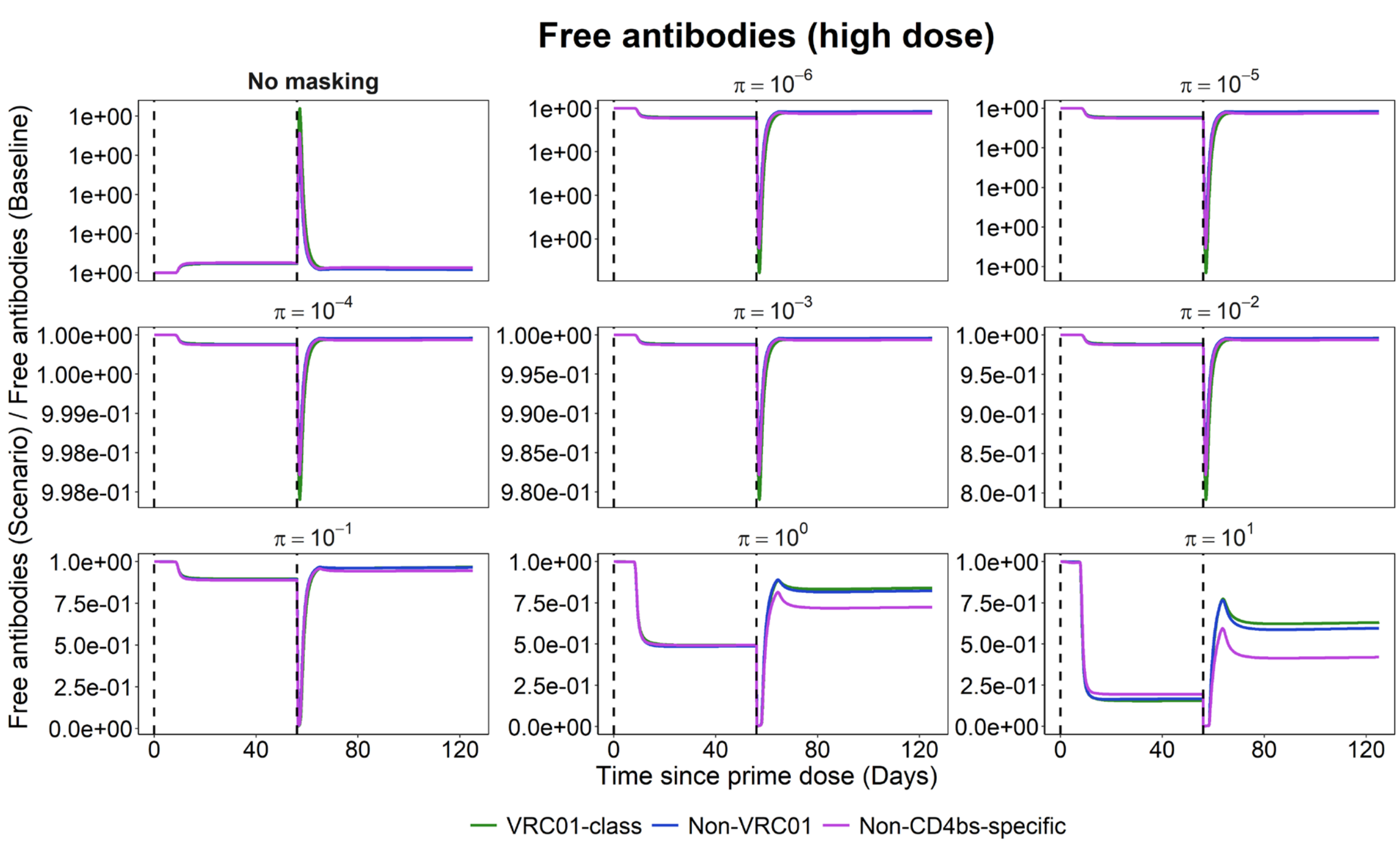

Figure SM14: Effect of antibody masking on vaccine-induced antibody dynamics. Ratio of antibody levels for different values of the immunogen–antibody binding rate (π) relative to the antibody level at $\pi = 10^{-7}$ (baseline).

bioRxiv preprint doi: https://doi.org/10.64898/2026.10.14.759230; this version posted October 20, 2026. The copyright holder for this preprint (which was not certified by peer review) is the author/funder, who has granted bioRxiv a license to display the preprint in perpetuity. It is made available under a CC-BY 4.0 International license.

Table SM2. Estimated population parameters across fixed antibody-immunogen binding rates. Values are population estimates (RSE, %). Each column corresponds to a model fitted with $\log_{10}(\pi)$ fixed at the indicated value. Parameters prefixed with $\log_{10}$ were estimated on the logarithmic scale; RSEs are reported on the same scale. NaN indicates that the RSE could not be evaluated.

Parameter	$\pi = 10^{-7}$	$\pi = 10^{-1}$	$\pi = 1$	$\pi = 10$
θ_a	1.2×10^{-3} (126)	7.1×10^{-5} (87.1)	9.8×10^{-5} (NaN)	1.4×10^{-4} (39.3)
θ_b	0.023 (162)	0.36 (26.7)	0.29 (23.3)	0.19 (53.4)
$\log_{10}(\zeta_m)$	1.96 (1.04)	2.11 (0.317)	2.05 (0.532)	2.09 (0.766)
$\log_{10}(\gamma_n)$	−11.03 (1.01)	−10.99 (1.12)	−10.79 (2.61)	−10.24 (1.98)
$\log_{10}(\gamma_{m1})$	−0.85 (5.08)	−0.91 (0.473)	−0.82 (2.37)	−0.83 (3.36)
$\log_{10}(\gamma_{m2})$	−0.33 (19.5)	−0.63 (NaN)	−0.54 (4.50)	−0.54 (6.61)
$\log_{10}(\gamma_{m3})$	−0.14 (35.1)	−0.17 (11.2)	−0.089 (32.6)	−0.058 (51.7)
δ_M	0.026 (8.72)	0.027 (8.58)	0.026 (8.64)	0.027 (8.64)
δ_l	8.0×10^{-4} (NaN)	6.9×10^{-4} (34.6)	8.1×10^{-4} (NaN)	1.0×10^{-3} (NaN)
$\log_{10}(K_{g1})$	7.81 (6.45)	6.84 (6.64)	7.09 (10.3)	6.54 (8.49)
$\log_{10}(K_{g2})$	3.97 (NaN)	5.67 (14.5)	5.52 (47.0)	3.02 (75.4)
$\log_{10}(K_{g3})$	7.96 (25.5)	7.81 (7.15)	8.19 (16.9)	8.22 (10.6)
$\log_{10}(\delta_{11})$	−2.39 (6.04)	−2.31 (3.31)	−2.39 (4.33)	−2.44 (3.42)
$\log_{10}(\delta_{12})$	−3.45 (5.78)	−2.96 (8.17)	−3.03 (20.7)	−3.08 (14.2)
$\log_{10}(\delta_{22})$	−1.58 (7.19)	−1.43 (5.33)	−1.47 (6.19)	−1.57 (5.38)
$\log_{10}(\delta_{13})$	−3.19 (32.4)	−3.07 (8.47)	−3.28 (15.6)	−3.23 (11.3)
$\log_{10}(\delta_{23})$	−2.34 (28.1)	−2.28 (13.0)	−2.44 (20.7)	−2.28 (15.2)
$\log_{10}(\delta_{33})$	−1.08 (11.3)	−0.93 (7.53)	−1.01 (9.95)	−1.04 (7.73)
$\log_{10}(\Psi)$	16.55 (0.574)	16.56 (0.348)	16.55 (0.607)	16.48 (0.362)

bioRxiv preprint doi: https://doi.org/10.64898/2026.10.14.759230; this version posted October 20, 2026. The copyright holder for this preprint (which was not certified by peer review) is the author/funder, who has granted bioRxiv a license to display the preprint in perpetuity. It is made available under a CC-BY 4.0 International license.

References

1. R. A. Manz, A. Thiel, A. Radbruch, Lifetime of plasma cells in the bone marrow. *Nature 1997 388:6638* 388, 133–134 (1997).
2. B. T. Mayer, *et al.*, Impact of LS Mutation on Pharmacokinetics of Preventive HIV Broadly Neutralizing Monoclonal Antibodies: A Cross-Protocol Analysis of 16 Clinical Trials in People without HIV. *Pharmaceutics* 16, 594 (2024).
3. E. D. Lobo, R. J. Hansen, J. P. Balthasar, Antibody Pharmacokinetics and Pharmacodynamics. *J. Pharm. Sci.* 93, 2645–2668 (2004).
4. J. P. Davda, M. G. Dodds, M. A. Gibbs, W. Wisdom, J. P. Gibbs, A model-based meta-analysis of monoclonal antibody pharmacokinetics to guide optimal first-in-human study design. *MAbs* 6, 1094–1102 (2014).
5. G. D. Victora, M. C. Nussenzweig, Germinal centers. *Annu. Rev. Immunol.* 30, 429–457 (2012).
6. A. D. Gitlin, Z. Shulman, M. C. Nussenzweig, Clonal selection in the germinal centre by regulated proliferation and hypermutation. *Nature 2014 509:7502* 509, 637–640 (2014).
7. C. Keşmir, R. J. De Boer, A Mathematical Model on Germinal Center Kinetics and Termination. *The Journal of Immunology* 163, 2463–2469 (1999).
8. A. S. Perelson, A. U. Neumann, M. Markowitz, J. M. Leonard, D. D. Ho, HIV-1 Dynamics in Vivo: Virion Clearance Rate, Infected Cell Life-Span, and Viral Generation Time. *Source: Science, New Series* 271, 1582–1586 (1996).
9. B. P. O'Connor, *et al.*, BCMA Is Essential for the Survival of Long-lived Bone Marrow Plasma Cells. *Journal of Experimental Medicine* 199, 91–98 (2004).
10. H. C. Allen, P. Sharma, Histology, Plasma Cells. *StatPearls* (2022).
11. M. K. Slifka, R. Ahmed, Long-term antibody production is sustained by antibody-secreting cells in the bone marrow following acute viral infection. *Ann. N. Y. Acad. Sci.* 797, 166–176 (1996).
12. I. C. M. MacLennan, Germinal centers. *Annu. Rev. Immunol.* 12, 117–139 (1994).
